# Heterogeneous grain-scale response in ferroic polycrystals under electric field

**DOI:** 10.1038/srep22820

**Published:** 2016-03-09

**Authors:** John E. Daniels, Marta Majkut, Qingua Cao, Søren Schmidt, Jon Wright, Wook Jo, Jette Oddershede

**Affiliations:** 1 School of Materials Science and Engineering, UNSW Australia, Sydney NSW 2052, Australia; 2 NEXMAP, DTU Physics, 2800 Kgs. Lyngby, Denmark; 3 European Synchrotron Radiation Facility, Grenoble 38000, France; 4 School of Mechanical and Advanced Materials Engineering, UNIST, Ulsan, Republic of Korea

## Abstract

Understanding coupling of ferroic properties over grain boundaries and within clusters of grains in polycrystalline materials is hindered due to a lack of direct experimental methods to probe the behaviour of individual grains in the bulk of a material. Here, a variant of three-dimensional X-ray diffraction (3D-XRD) is used to resolve the non-180° ferroelectric domain switching strain components of 191 grains from the bulk of a polycrystalline electro-ceramic that has undergone an electric-field-induced phase transformation. It is found that while the orientation of a given grain relative to the field direction has a significant influence on the phase and resultant domain texture, there are large deviations from the average behaviour at the grain scale. It is suggested that these deviations arise from local strain and electric field neighbourhoods being highly heterogeneous within the bulk polycrystal. Additionally, the minimisation of electrostatic potentials at the grain boundaries due to interacting ferroelectric domains must also be considered. It is found that the local grain-scale deviations average out over approximately 10–20 grains. These results provide unique insight into the grain-scale interactions of ferroic materials and will be of value for future efforts to comprehensively model these and related materials at that length-scale.

Piezoelectric materials offer the ability to directly couple electrical charge and mechanical strain, and have a vast range of technological applications. Recent progress towards the development of lead-free piezoelectric materials to meet new legislative requirements^1^ has directed detailed investigations of the strain mechanisms in both existing lead-containing and promising lead-free systems.

The magnitude of electric-field-induced strain possible in a given material is often a limiting factor for device design, particularly for actuators. Lead-based and lead-free single crystal materials have been shown to exhibit very large reversible strains under external electric field^2^,^3^, however, difficulties and costs associated with crystal growth limit their applicability. Ceramic materials offer significant advantages in terms of processing, however, the total strain achievable in ceramics is much less than that obtainable in single crystals optimised for high strain applications. The limitation of achievable strain in ceramic materials results from the intergranular constraint of the polycrystal, which restricts the large anisotropic responses of the individual grains. Despite this, thorough understanding of the intergranular responses of these materials is lacking, primarily due to experimental difficulties associated with probing polycrystalline materials at this length-scale.

In this study, a variant of the grain-resolved scattering method, 3D-XRD, where the non-180° ferroelectric domain switching strain response of grains can be probed independently^4^, is applied to show the phase and domain structure evolution of individual grain orientations within a bulk polycrystalline electro-ceramic under applied electric field. Such investigations provide unique information on the grain-scale electro-mechanical coupling in ceramics and are of benefit to the future engineering of high-strain actuators. Additionally, the information provided by the present measurements is applicable to a broad range of materials that strain via ferroelastic phase transformations and/or subsequent domain wall motion, such as superelastics and shape memory alloys.

The material chosen for the present study is (0.82)Bi_0.5_Na_0.5_TiO_3_ – (0.18)Bi_0.5_K_0.5_TiO_3_. Bi_0.5_Na_0.5_TiO_3_ (BNT) based ceramics produced in solid solution with (among others) either BaTiO_3_ (BT)^5^, Bi_0.5_K_0.5_TiO_3_ (BKT)^6^, K_0.5_Na_0.5_NbO_3_ (KNN)^7^ or combinations thereof^8^,^9^,^10^,^11^,^12^,^13^ have been shown to possess usable electric-field-induced strains of the order of 0.4%^14^. For given compositions, an initial pseudo-cubic non-ferroic structure has been observed to transform to a ferroic state with majority tetragonal^15^, or a phase mixture of tetragonal and rhombohedral symmetries^16^. The process is irreversible under some conditions, as in the present experiments, and reversible at others^17^. The driving force for electric-field-induced transformations in a free single crystal is often attributed to a flattening of the free energy profile in the vicinity of phase boundaries^18^. Thus, the external field in this case can drive or rotate the ferroelectric polar axis within the fixed crystal orientation to align with the external field vector, resulting in multiple phase symmetries. This phase transformation can be the source of either the large useable strain, or the large remnant strain in the cases of a reversible and irreversible transformation, respectively.

## Results and Discussion

**Figure 1:**
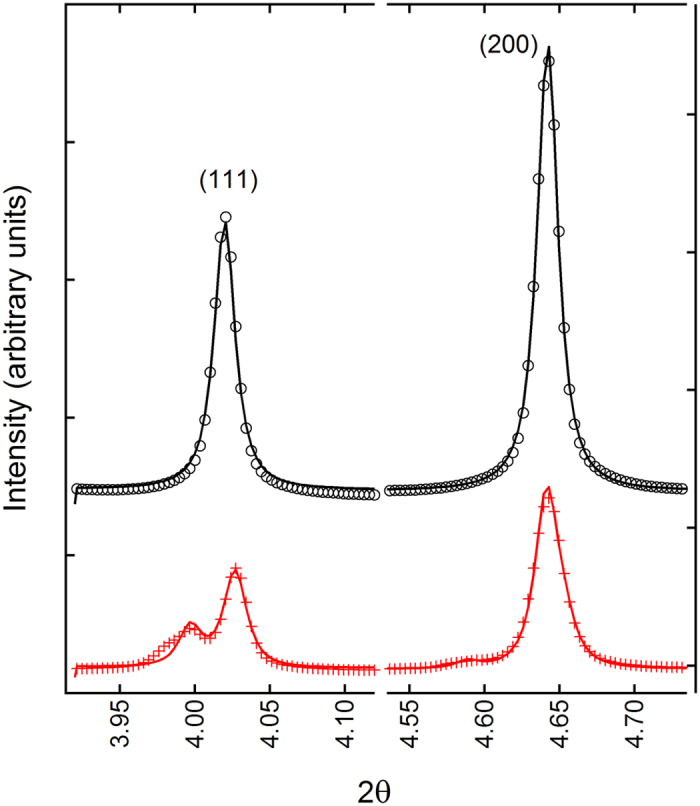
Powder average (111) and (002) diffraction peaks before (top) and after (bottom) the application of an electric field. The as-processed structure has single symmetric diffraction peaks expected from the pseudo-cubic phase. After the application of electric field, the sample has transformed to majority rhombohedral symmetry (splitting of the 111). The peak indices are for the pseudo-cubic perovskite structure.

Selected regions of the resulting powder diffraction patterns are shown above in Fig. 1. In the as-processed state, no peak splitting or peak shape asymmetry is observed, confirming that the materials exists in the “pseudo-cubic” state, which is often observed in this and related compositions^19^. The refined lattice parameter of this pseudo-cubic phase is found to be 3.905 Å. Once poled, the material shows a majority rhombohedral structure, with R3c lattice parameters of a = 5.510 Å and c = 13.609 Å, or in the alternatively rhombohedral setting, a = b = c = 3.905 Å, and α = β = γ = 89.686°.

The 3D-XRD method allows for the indexing of many individual grains from a bulk polycrystalline material and provides direct access to grain orientation resolved information. To extract this information, the sequence of diffraction images were processed using the Fable software package^20^. The original pseudo-cubic orientation of the grains in the poled state was determined in the indexing step using GrainSpotter^21^. By applying a high completeness cut-off in terms of the number of observed reflections compared to expected reflections, the indexing can be limited to the grains that are located within the central part of the sample, which is illuminated throughout the rotation. Within the scattering volume sampled, a total of 507 grains were indexed.

In the rhombohedral structure, four possible domain variants exist with a unique spontaneous strain axis relative to the grain orientation. In order to fit the volume fractions 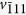, 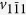, 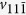 and *v*_111_ of these domains within each grain, individual *111* reflection sets assigned to the grain were extracted from the data volume. After correcting the intensities for the Lorentz and polarisation factors, the grain volume and the associated errors were derived. Each *111* reflection was then integrated into a radial profile along 2θ, and the radial intensity splitting was used to fit the domain volume fractions in a weighted least squares procedure with the constraint 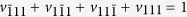 as outlined in detail by Oddershede *et al.*^4^. The diffraction experiment covered a total angular range, ω, of 320°, including Friedel pairs this gives up to 16 observations of the *111* reflection family for each grain. After fitting the domain volume fractions, the strain parallel to the electric field vector resulting from the domain texture of the induced phase, ε_p_, and the associated errors were determined^4^,^22^. In the fitting procedure and subsequent analysis, only 191 grains that satisfied the following criteria were considered: 1) at least one unique observation of a *111* reflection for each domain variant, 2) a minimum of eight observations of all possible *111* reflections in total, 3) intensity consistency between all *111* reflections of a given grain, and 4) absolute strain errors less than 0.04%. These criteria eliminate the possibility of experimental errors caused by reflection overlap and/or the associated grains rotating in and out of the illuminated volume.

**Figure 2:**
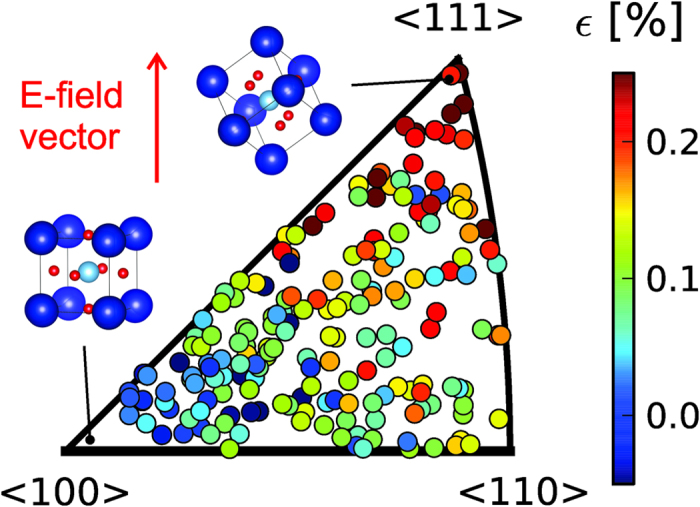
Inverse pole figure of indexed grain orientations. Each marker represents a single grain in the cross section of material intersected by the X-ray beam. The position on the plot represents the pseudo-cubic direction of the grain which lies parallel to the electric field vector, e.g. a grain in the bottom left of the figure has a <001> direction parallel to the electric field, while a grain in the top right has a <111> direction parallel to the electric field (see inset unit cells). The marker colour represents the calculated the non-180° ferroelectric domain switching strain along the field direction.

The initial indexing of the total 507 grains partially or fully sampled confirmed, via a Mackenzie type analysis^23^, that the measured grains represent a random distribution of orientations, as expected from the ceramic processing technique used. Figure 2 shows the orientations of the 191 grains, which satisfied the above stated consistency criteria, plotted in an inverse pole figure. Each marker represents a single grain, and its position in the diagram is representative of the pseudo-cubic direction of the grain that lies parallel to the applied electric field vector. This orientation relationship is highlighted with the prototypical ABO_3_ perovskite unit cell shown by the inset diagrams of Fig. 2. The <100> corner of the inverse pole figure is relatively empty. In fact, no grains with a <001> direction within approximately 7° of the electric field vector satisfied the consistency criteria outlined above for fitting of rhombohedral domain volume fractions, despite several being initially indexed. Upon inspection of the raw diffraction data, it is concluded that this is most likely due to these grains having a significant volume fraction that either remains in the pseudo-cubic state or transforms partially to a tetragonal or other lower symmetry structure. This is consistent with the fact that related materials have field-induced symmetries that are highly sensitive to small compositional changes. For example, it is observed in the BNT-x%BT system that at a stoichiometry of approximately x = 7 the system transforms to a single tetragonal phase^15^, while in nominal x = 6 compositions, a mixed phase system of tetragonal and rhombohedral symmetries exists^16^. It is therefore likely the current composition sits within a region of the phase diagram just to one side of a mixed phase region and a very slight adjustment to the stoichiometry could result in grains close to the <100> corner of the pole figure transforming to tetragonal or rhombohedral symmetry.

From Fig. 2 it’s clear that there is a general trend towards maximum and minimum non-180° ferroelectric domain switching strain values occurring at grain orientations with a <111> and <100> direction lying close to the electric field vector, respectively. This is as expected, since the maximum domain switching strain results when a variant of a grains <111> directions aligns closely to the electric field vector giving a theoretical maximum strain of ε_p_ = 0.547% if the grain was in the saturated monodomain state. Grains with a <100> direction aligned closely to the electric field direction have the minimum strain of 0, as all <111> directions have equal angles to the applied field and a zero spontaneous strain component regardless of the domain populations. However, in addition to the observed first order correlation between grain orientation and strain there are significant variations, or second order perturbations within groups of grains of similar orientations. More interestingly, there are in fact grains that show negative non-180° ferroelectric domain switching strain components along the field direction.

**Figure 3:**
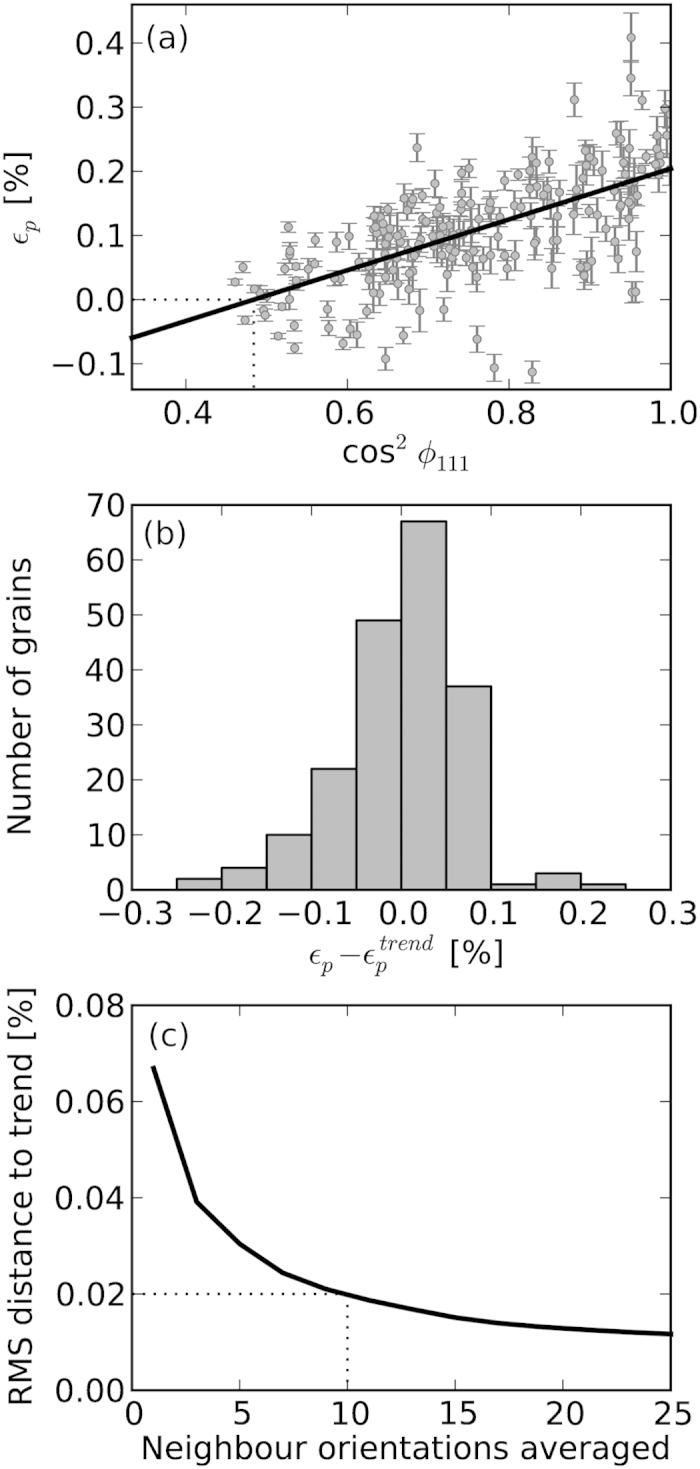
(**a**) domain switching strains along the poling direction as a function of cos^2^ϕ_111_, where ϕ_111_ is the misorientation between the electric field vector and the closest <111> direction in the grain, (**b**) distribution of actual domain switching strains away from average behaviour, and (**c**) RMS distance to the trend line in (**a**) as a function of group size when averaging over groups of grains with similar orientations. Based on this it is concluded that the local grain-scale deviations average out over approximately 10–20 grains.

To highlight these variations, the dimensionality of the representation is reduced by showing in Fig. 3(a) the measured (grey with error bars) non-180° ferroelectric domain switching strains resolved in the direction of the electric-field vector for each grain as a function of cos^2^ϕ_111_, where ϕ_111_ is the misorientation between the electric field vector and the closest <111> direction in the given grain. Here it is clear that individual grain behaviour varies significantly, and is not dictated solely by the orientation of the grain relative to the applied field vector. To more clearly observe the magnitude of the deviation away from average behaviour, the difference between the calculated strain, and that expected from average behaviour is shown in Fig. 3(b).

These deviations in resultant non-180° ferroelectric domain switching strain in the field-induced phase must be accommodated to some extent by lattice strains of the grains. Such lattice strains may arise from a combination of the intrinsic piezoelectric effect and elastic compliance strains^24^, both of which are highly anisotropic with respect to the crystallographic direction. Deconvolution of these two mechanisms is not possible with the current data, however, future extensions to X-ray microscopy methods that allow direct probing of the strain of individual domains may facilitate this^25^. The outlier grains on the strain magnitude distribution likely experience very large intergranular stresses with their neighbours, and thus, are perhaps sources of crack initiation which may lead to reduced fatigue lifetimes upon cycling. Detailed analysis of these individual neighbour interactions is not possible in the current study, as the combination of grain size and detector resolution does not allow for reconstruction of the spatial distribution of grains, but investigations of this type may become possible in the future by expanding on existing grain mapping techniques^26^,^27^,^28^.

The present results provide quantitative measures of the local grain-scale deviations from the bulk average strain response of the piezoelectric ceramic material. The microstructural origins of this grain-scale heterogeneity lie in several possible areas which are likely acting in parallel. Firstly, the highly anisotropic piezoelectric response of these materials will cause the local stress environment of each grain to be different. The domain switching response of the individual grain is thus limited (or enhanced) by the total piezoelectric strain response of the surrounding grains. Secondly, dielectric anisotropy creates inhomogeneous field magnitudes at any point within a polycrystalline material. These inhomogeneous fields have been suggested to cause distributions of domain switching times in related piezoelectric ceramics^29^. Finally, it is often observed in ferroelectric/ferroelastic polycrystals that domain morphologies differ significantly at grain boundaries and in some cases propagate through the boundary^30^. This is a result of the very high electro-static energy resulting from non head-to-tail domain configurations that would inevitably form at grain boundaries between two randomly oriented grains with random domain structures. It may be that this electrostatic energy outweighs the elastic energy required to compensate for the non-uniform strain resulting from continuity of domain walls between grains. Such domain sharing may lead to grain-scale collective dynamics in bulk materials, similar to that observed in polycrystalline films^31^,^32^. The total system energy will therefore include a combination of electrostatic potential at the grain boundaries and the elastic strain energy associated with compensating the anisotropic non-180° ferroelectric domain switching strain during the phase transformation. The requirement for balance between these effects to minimise the global energy of the polycrystalline system ensures that polycrystalline ferroelectrics are highly inhomogeneous at the grain scale.

A critical question that arises from such a result is at what length-scale, or over how many grains, do these local deviations average out? This length-scale will be particularly important when considering the validity of modelling efforts of bulk materials at the granular and sub-granular scale. In order to quantify this, the measured grains are grouped into nearest orientation neighbours and the groups then compared to the overall trend behaviour for all grains. This is displayed in Fig. 3(c) as the RMS distance to overall trend as a function of grouped grain neighbours. When just single isolated grains are taken, the distribution has a RMS difference to the mean of 0.067%. It can be seen that the difference between the group of grains and the general trend reduces rapidly as the total number of grains included increases. Here, it appears as though the grain clusters approach the bulk average behaviour (i.e. RMS value asymptotes) at cluster sizes between 10 and 20 grains.

In summary, it is shown that in a polycrystalline ceramic of (0.82)Bi_0.5_Na_0.5_TiO_3_ – (0.18)Bi_0.5_K_0.5_TiO_3_, the grain orientation relative to the applied electric field influences the resulting phase and domain structure of the electric-field-induced phase. In this particular composition, most grains were found to transform to a rhombohedral symmetry, while grains with a <100> direction within 7**°** of the applied field vector displayed a significant volume fraction that either remained in the cubic state or transformed partially to a tetragonal or other lower symmetry structure. Of the grains that transformed to a rhombohedral symmetry, a variation in the resulting domain texture existed which had the trend from less to more saturated when the <100> and <111> directions of a grain were aligned with the field vector, respectively. However, significant deviations in the magnitude of the response exist at the grain scale, as evidenced by the large variation in resulting domain texture strains observed for grains of similar orientations. The length-scale on which these local variations average out is likely of the order of 10–20 grains. The origin of these deviations is suggested to result from complex interactions of grain-neighbour strain magnitudes, electric field magnitude inhomogeneities and the interaction of ferroic domains at the grain boundaries. These results are of critical importance when considering the validity of grain-scale modelling efforts, and provide additional considerations in the design of novel electro-mechanical materials.

## Methods

Samples of (0.82)Bi_0.5_Na_0.5_TiO_3_ – (0.18)Bi_0.5_K_0.5_TiO_3_ were produced by the mixed oxide route. Details of the synthesis method can be found elsewhere^33^. The resulting grain size of the samples was approximately 3–5 μm. A sample was cut and polished into a rectangular shape of dimensions 100 × 200 × 90 μm^3^. Gold electrodes were sputtered onto two opposing 100 × 200 μm^2^ surfaces. The sample was then placed with an electrode surface in contact with a brass pin and electrically contacted using silver paint. A top electrode wire was connected using silver paint and the sample encapsulated in a 1 mm diameter Kapton tube filled with silicone oil. Such a setup allows the application of high electric fields without the risk of dielectric breakdown^34^.

X-ray diffraction experiments were performed at beamline ID11 of the European Synchrotron Radiation Facility. A beam energy of 78.40 keV and dimensions 50 μm in width × 5 μm in height was produced at the sample position. The planar beam transmits through the sample such that it interacts with a limited number of grains creating “spotty” diffraction images. The sample was then rotated around and axis perpendicular to the incident beam, ω, with data collected over 2 × 160° in 0.25° integration angles. A schematic diagram of the 3D-XRD setup can be found in Oddershede^4^. For more details of the data collection strategy see Poulsen^35^. After the collection of an initial data set in the as-processed state, the sample was electrically poled with a field of 4 kV/mm and a subsequent data set collected.

Unit cell parameters for the as-processed and poled materials were found from full pattern refinements using Topas V4.1. The powder diffraction data were generated by creating a sum of all diffraction images recorded as a function of sample rotation angle, ω, and then integrating the resulting 2D diffraction patterns in azimuthal angle, η.

## Additional Information

**How to cite this article**: Daniels, J. E. *et al.* Heterogeneous grain-scale response in ferroic polycrystals under electric field. *Sci. Rep.* **6**, 22820; doi: 10.1038/srep22820 (2016).
